# Beyond burnout: occupational risk pathways linking stress, sleep, and multisystem morbidity in correctional officers

**DOI:** 10.3389/fpubh.2026.1901314

**Published:** 2026-08-04

**Authors:** Shiqi Sun, Peng Xing, Yi Liu, Dongming Jia

**Affiliations:** 1 Zhejiang Police Vocational College, Hangzhou, Zhejiang, China; 2 Taihu Institute of Addiction Rehabilitation, Suzhou, Jiangsu, China

**Keywords:** burnout, cardiometabolic risk, correctional officers, occupational risk pathways, public health, total worker health^®^

## Abstract

**Background:**

Correctional officers work in security-intensive environments characterized by organizational strain, threat exposure, shift work, staffing shortages, and restricted autonomy. Psychological outcomes have been most studied, yet sleep disruption, cardiometabolic risk, musculoskeletal pain, and fatigue are accumulating but often examined separately, obscuring multisystem occupational morbidity and prevention priorities.

**Objective:**

To synthesize mental and emerging physical health risks among correctional officers using an integrated occupational risk pathways framework linking upstream exposures, bridging mechanisms, downstream outcomes, and intervention levers.

**Methods:**

A structured narrative review informed by SANRA was conducted. Literature was identified from PubMed, Web of Science, Scopus, PsycINFO, and CNKI through February 2026. Two independent reviewers screened 405 records on title and abstract; 127 records proceeded to full-text review, of which 58 were excluded and 69 sources were retained for narrative synthesis. Evidence domains were classified as established, moderate, emerging, or plausible.

**Results:**

The synthesis indicates substantial mental health burden (burnout, depression, anxiety, PTSD, moral injury, suicide risk) and emerging physical health evidence (cardiometabolic risk, musculoskeletal pain, fatigue). Sleep disruption, chronic stress, allostatic load, and hypervigilance emerge as central bridging mechanisms. Individual-focused interventions may have limited durability when organizational exposures persist; Total Worker Health-informed strategies integrating staffing, schedule reform, procedural justice, sleep and fatigue management, and surveillance are more consistent with multi-pathway risk.

**Conclusion:**

Correctional officer health should be treated as a public health and occupational safety issue rather than individual resilience failure. Future research should prioritize longitudinal cohorts, objective sleep and biomarker assessment, and rigorous evaluation of organizational interventions.

## 1 Introduction

Correctional officers (COs), also referred to as prison officers, jail officers, or correctional staff, are an essential but comparatively underexamined occupational group in public health and occupational health research (1, 2). Their work is performed in closed, security-oriented institutions characterized by persistent interpersonal threat, rigid hierarchy, limited decision latitude, repeated exposure to conflict, and responsibility for maintaining safety under conditions of social and institutional constraint (3, 4). Unlike many public safety occupations in which critical incidents may be episodic, correctional work often combines chronic organizational stressors with recurrent acute events over prolonged service periods (5).

Three gaps motivate the present review. First, the empirical literature remains uneven. Mental health outcomes among COs, including burnout, depression, anxiety, post-traumatic stress disorder (PTSD), and suicide risk, are comparatively well described (6, 7). By contrast, physical health outcomes, including sleep disruption, cardiometabolic risk, musculoskeletal pain, fatigue, and lifestyle-related deterioration, are accumulating but remain less integrated into correctional occupational health models (8–11). This unevenness has encouraged a fragmented literature in which mental and physical outcomes are often treated as separate domains rather than co-occurring manifestations of cumulative occupational strain.

Second, the field lacks a sufficiently explicit mechanistic framework. Shift work, violence exposure, mandatory overtime, organizational injustice, low autonomy, and occupational stigma are repeatedly described as risk factors, yet fewer studies specify how these exposures may operate through sleep disruption, chronic stress-system activation, hypervigilance, maladaptive coping, and physiological dysregulation to produce multisystem morbidity. A pathway perspective is needed because the same work conditions may contribute simultaneously to emotional exhaustion, trauma symptoms, hypertension, obesity, pain, and fatigue through shared mechanisms.

Third, the intervention literature remains misaligned with the structure of risk. Many interventions emphasize individual-level coping, resilience, mindfulness, or help-seeking. Such strategies may be useful, but their durability is likely limited when staffing shortages, unpredictable schedules, excessive overtime, poor supervisory support, and inadequate recovery remain unchanged (3, 12). A public health approach to correctional officer wellbeing therefore requires moving upstream: from crisis response and individual stress management toward integrated prevention, occupational safety, and organizational redesign.

To address these gaps, this review synthesizes the literature on mental and emerging physical health risks among COs through an integrated occupational risk pathways framework. The review has four aims: (1) to synthesize evidence on mental health burden and emerging physical health risks; (2) to organize findings according to upstream exposures, bridging mechanisms, and downstream outcomes; (3) to evaluate the implications of individual, peer/team, organizational, and integrated Total Worker Health (TWH) approaches; and (4) to propose a research and policy agenda for correctional occupational health. The central argument is that CO health burden should be understood not as a set of isolated disorders, but as a multisystem occupational morbidity pattern generated by interacting organizational, psychosocial, behavioral, and physiological pathways.

Recent scoping reviews have mapped psychological health strategies and integrated safety promotion among correctional workers (1, 2), yet few syntheses link mental and emerging physical health outcomes through explicit bridging mechanisms or stratify evidence maturity across pathways. This review addresses that gap by organizing correctional officer health evidence within an integrated occupational risk pathways framework and distinguishing established from emerging domains.

## 2 Correctional officer health literature and rationale for synthesis

Research on correctional officer (CO) health has expanded substantially over the past decade, yet remains uneven across outcome domains. Mental health burden—including burnout, depression, anxiety, PTSD, moral injury, and suicide risk—is comparatively well documented across North American, European, Latin American, and Chinese correctional systems (6, 7, 13–16). Chinese prison police subculture and regulatory context have also been analyzed as upstream organizational influences (17). Organizational stressors such as shift work, violence exposure, mandatory overtime, low autonomy, and perceived injustice are repeatedly associated with psychological distress and burnout (13, 14, 18–25). Help-seeking barriers, occupational stigma, and masculine institutional norms further constrain access to care (26–29).

Physical and behavioral health evidence is less integrated but increasingly visible. Studies report elevated sleep disruption, cardiometabolic risk, musculoskeletal pain, tobacco and alcohol use, and fatigue among COs (30–39). Sleep problems, in particular, appear to bridge occupational exposures and both psychological and physiological outcomes (40, 41). Biomarker and wearable-sensor assessments remain sparse, although salivary cortisol, heart rate variability, and pilot sensor studies suggest measurable physiological dysregulation in correctional workforces (42–44).

Prior reviews have mapped psychological health strategies, integrated safety promotion, organizational stressors, and wellbeing interventions among correctional workers (1, 2, 5, 12, 45). These syntheses provide essential orientation but typically treat mental and physical outcomes separately, offer limited mechanistic linkage across pathways, or focus on intervention catalogs rather than multisystem occupational morbidity. Scoping and narrative reviews in adjacent public safety literatures similarly emphasize individual resilience or single-outcome models (4, 46).

Accordingly, before presenting the methods and integrated framework developed for the present synthesis, this section summarizes the correctional officer health literature as an orienting background. Detailed pathway-specific evidence is developed in Sections 4–9, where upstream exposures, bridging mechanisms, downstream outcomes, intervention levers, and evidence maturity are examined systematically within the proposed occupational risk pathways model.

## 3 Methods

### 3.1 Review design and reporting orientation

This article is a structured narrative review and framework-oriented conceptual synthesis. A narrative approach was selected because the CO health literature spans heterogeneous study designs, measurement instruments, occupational systems, and outcome domains, making quantitative pooling neither feasible nor appropriate for the central aim of this article. The review was informed by the Scale for the Assessment of Narrative Review Articles (SANRA), particularly its emphasis on justification of importance, explicit aims, transparent literature identification, appropriate referencing, scientific reasoning, and balanced presentation (47, 48). The review was not prospectively registered, consistent with its narrative, framework-oriented design; a SANRA-based self-assessment was used to guide transparent quality reporting.

The structure below uses a topic-specific synthesis format appropriate to framework-oriented narrative reviews, organized around upstream exposures, bridging mechanisms, downstream outcomes, and intervention levers rather than an original-research IMRaD template (47, 48). The purpose of the transparent record-flow process was to support framework-oriented narrative synthesis rather than to produce an exhaustive systematic evidence map.

A registered systematic review with meta-analysis and formal GRADE certainty ratings was not undertaken because the primary objective was conceptual integration across heterogeneous outcome domains rather than estimation of pooled effect sizes for discrete exposures or interventions. The CO health literature spans cross-sectional surveys, qualitative studies, cohort reports, program evaluations, and policy analyses with non-comparable designs, instruments, and metrics, making quantitative synthesis inappropriate for the framework-building aim. Transparent dual screening, prespecified eligibility criteria, and a PRISMA-inspired record flow (Supplementary file S2) were nevertheless applied to reduce selection bias. Formal study-level risk-of-bias assessment and GRADE grading were reserved for future focused systematic updates addressing narrower questions—for example, intervention effectiveness for defined outcomes—while the present review used an interpretive established/moderate/emerging/plausible classification aligned with SANRA guidance for narrative synthesis (47, 48).

### 3.2 Literature identification and eligibility

Sources were identified through searches of PubMed, Web of Science, Scopus, and PsycINFO for English-language literature and the China National Knowledge Infrastructure (CNKI) for Chinese-language literature. Searches were updated through February 2026. Literature published from 2016 onward was emphasized to reflect the contemporary correctional work environment, while earlier studies were retained when they were foundational or conceptually important. Search strings combined terms for occupational role (e.g., correctional officer^*^, prison officer^*^, jail staff), exposures (e.g., occupational stress, shift work, violence exposure, organizational justice), outcomes (e.g., burnout, depression, anxiety, PTSD, sleep, cardiometabolic, musculoskeletal), and interventions (e.g., health promotion, Total Worker Health, peer support, resilience training). The PubMed strategy and study selection logic are provided in Supplementary file S1, and a PRISMA-inspired flow diagram adapted for structured narrative synthesis is provided in Supplementary file S2.

Eligible sources included peer-reviewed empirical studies using quantitative, qualitative, or mixed methods that reported mental health outcomes, physical health outcomes, behavioral health outcomes, or health promotion interventions among correctional, prison, or jail staff. Systematic reviews, scoping reviews, theoretical papers, and policy-oriented sources were retained when they informed synthesis or framework development. Records were excluded when they lacked correctional-officer-specific data or addressed other public safety groups without separable correctional staff findings.

### 3.3 Screening, synthesis, and framework construction

The search process identified 405 records for screening (303 English-language; 102 Chinese-language). Two reviewers (DJ and an independent second screener [SX], acknowledged below) independently screened titles and abstracts against prespecified eligibility criteria; disagreements at any screening stage were resolved through discussion. After title and abstract screening, 198 English-language and 80 Chinese-language records were excluded, leaving 127 records (105 English-language; 22 Chinese-language) for full-text assessment. Both reviewers conducted full-text review of all 127 records; 58 were excluded at this stage because they did not meet one or more eligibility criteria (see Section 3.2). The remaining 69 sources were retained for narrative synthesis. The second screener (SX) contributed to title, abstract, and full-text screening; she did not participate in evidence interpretation, framework development, or manuscript writing and is therefore acknowledged rather than listed as an author. Additional references cited in the manuscript draw on methodological, theoretical, and adjacent occupational health literatures and were identified through targeted backward citation searches of retained articles and pertinent prior reviews (Supplementary file S1). Sources were then organized into an occupational risk pathways matrix with four levels: upstream exposures, bridging mechanisms, downstream outcomes, and intervention levers. Initial domains were informed by established occupational health concepts, including TWH and allostatic load theory, and were refined iteratively as correctional officer-specific evidence reviewed (Table 1).

**Table 1:** Framework construction logic for the structured narrative synthesis.

Step	Procedure	Output
1. Literature mapping	Identify correctional officer studies and relevant occupational health frameworks across mental, physical, behavioral, and organizational domains.	Evidence domains for synthesis.
2. Pathway extraction	Map occupational exposures, candidate mediators, health outcomes, and intervention targets.	Preliminary exposure–mechanism–outcome matrix.
3. Evidence maturity classification	Classify each domain as established, moderate, emerging, or plausible based on the volume and specificity of CO evidence.	Interpretive weighting of conclusions.
4. Framework integration	Integrate correctional evidence with TWH and allostatic load concepts.	Final occupational risk pathways framework.
5. Research and policy translation	Identify modifiable intervention levers at individual, peer/team, organizational, and integrated–system levels.	Research agenda and practice implications.

For interpretive transparency, evidence domains were classified as established, moderate, emerging, or plausible. Established domains were supported by multiple studies or prior reviews in CO populations. Moderate domains were supported by several empirical studies but with heterogeneity in methods or settings. Emerging domains had limited CO-specific evidence but growing empirical support. Plausible domains were biologically or theoretically grounded but supported mainly by adjacent occupational health literatures or limited correctional samples. Two authors independently assigned evidence domains to these categories using the criteria above, and disagreements were resolved by discussion. This classification is not equivalent to formal GRADE certainty assessment; it is used to clarify the maturity of evidence in a narrative framework synthesis.

## 4 An integrated occupational risk pathways framework

**Figure 1:**
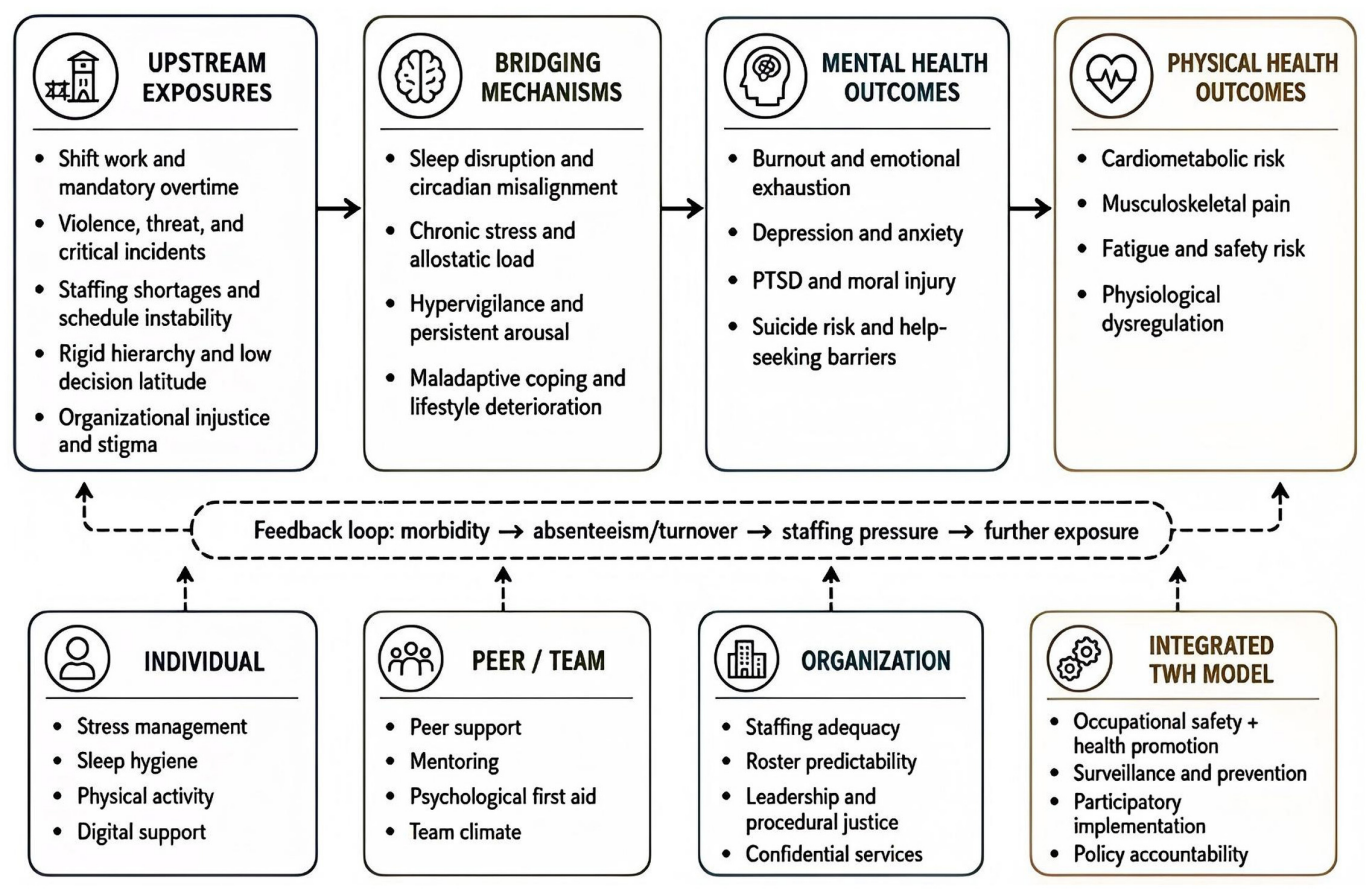
Integrated occupational risk pathways framework for correctional officer health. Upstream occupational exposures comprise structural/organizational conditions (shift work and rotating schedules, mandatory overtime, staffing shortages, schedule instability, rigid hierarchy, low autonomy, and organizational injustice) and psychosocial/interpersonal conditions (violence and threat exposure, critical incidents, role conflict, moral strain, occupational devaluation, and stigma). These exposures operate through bridging mechanisms, including sleep disruption and circadian misalignment, chronic stress and allostatic load, hypervigilance and persistent arousal, and maladaptive coping with lifestyle deterioration, generating downstream multisystem morbidity. Mental health outcomes include burnout, emotional exhaustion, depression, anxiety, PTSD, moral injury, suicide risk, and help-seeking barriers. Physical and behavioral outcomes include cardiometabolic risk, musculoskeletal pain, fatigue and safety risk, and physiological dysregulation. Intervention levers act at four levels: individual, peer/team, organizational, and integrated Total Worker Health^®^ model. Solid arrows indicate core pathways supported by converging evidence; dashed arrows indicate intervention levers and feedback loops (morbidity -> absenteeism/turnover -> staffing pressure -> further exposure) requiring further evaluation.

The proposed framework conceptualizes CO health as the result of interacting pathways rather than isolated occupational hazards (Figure 1). Upstream exposures include structural and organizational conditions, such as shift work, mandatory overtime, staffing shortages, schedule instability, rigid hierarchy, low autonomy, poor leadership, and organizational injustice. Psychosocial and interpersonal exposures include violence and threat exposure, critical incidents, emotional labor, role conflict, moral strain, occupational devaluation, and stigma.

These exposures may operate through bridging mechanisms, including sleep disruption and circadian misalignment, chronic stress and allostatic load, hypervigilance, maladaptive coping, and lifestyle deterioration. Downstream outcomes include mental health problems, such as burnout, depression, anxiety, PTSD, moral injury, and suicide risk, as well as physical and behavioral health outcomes, such as cardiometabolic risk, musculoskeletal pain, fatigue, and physiological dysregulation. The framework also includes feedback loops. Health deterioration may increase absenteeism, presenteeism, early retirement, and turnover, which may further worsen staffing shortages and increase burden on remaining officers (Table 2).

**Table 2:** Occupational exposures, mechanisms, outcomes, and intervention implications.

Domain	Main components	Intervention implication
Structural exposures	Shift work, mandatory overtime, staffing shortages, schedule instability, low autonomy, rigid hierarchy.	Prioritize roster predictability, staffing adequacy, recovery time, and procedural fairness.
Psychosocial exposures	Violence exposure, critical incidents, emotional labor, moral strain, occupational stigma.	Develop trauma–informed supervision, peer support, confidential services, and moral–injury–aware support.
Bridging mechanisms	Sleep disruption, chronic stress, allostatic load, hypervigilance, maladaptive coping.	Measure sleep, fatigue, stress physiology, and coping behaviors as pathway indicators.
Mental health outcomes	Burnout, depression, anxiety, PTSD, moral injury, suicide risk, help–seeking barriers.	Combine early detection, confidential care, stigma reduction, and leadership accountability.
Physical outcomes	Cardiometabolic risk, musculoskeletal pain, fatigue, physiological dysregulation.	Integrate medical surveillance, ergonomic prevention, workplace activity and nutrition supports, and fatigue management.
Integrated model	TWH–informed prevention connecting occupational safety with health promotion.	Move from individual resilience programs to multilevel systems intervention.

## 5 Upstream occupational exposures

### 5.1 Structural and organizational exposures

Correctional work is shaped by structural conditions that can generate chronic strain. Shift work and overnight duty disrupt circadian rhythms and family routines, impair recovery, and contribute to fatigue and emotional dysregulation (49, 50). Qualitative work further describes layered emotionality and identity construction as enduring features of correctional officer occupational culture (51). Staffing shortages and mandatory overtime are increasingly described as systemic problems, forcing officers to work extended hours and reducing opportunities for sleep, family life, exercise, and recovery (49, 52, 53). Schedule instability can further reduce perceived control and increase anticipatory stress.

Rigid hierarchy, strong security protocols, and limited decision latitude are also central occupational conditions. COs must enforce institutional rules while navigating complex interpersonal situations, often with limited autonomy and under high accountability. Perceived unfairness in scheduling, promotion, discipline, and supervision may intensify burnout and psychological distress (23–25). These organizational exposures matter because they are modifiable: unlike individual vulnerability, staffing policy, roster design, leadership training, and procedural justice are institutional levers.

### 5.2 Psychosocial and interpersonal exposures

Violence and threat exposure are defining features of correctional work. Officers may experience verbal abuse, physical assault, hostage threats, exposure to self-harm or suicide attempts, and recurrent anticipation of violence (1, 54). Chronic hypervigilance may be adaptive within the institution but becomes harmful when it persists off duty and impairs sleep, emotional regulation, and family relationships. Role conflict and moral strain may also occur when officers must reconcile custody, care, rehabilitation, security, and human service expectations under institutional constraints (55, 56).

Occupational devaluation and stigma form an additional psychosocial pathway. COs are often less publicly recognized than other public safety workers, and institutional cultures may normalize distress, discourage help-seeking, or frame mental health difficulties as weakness (26–28, 57, 58). These dynamics are important because they may reduce utilization of employee assistance programs and undermine early intervention. Work-family conflict represents a further psychosocial pathway: the demands of shift work, overtime, and emotional burden from the job can spill over into family life, straining relationships and reducing recovery opportunities at home (41, 59, 60). Female officers and those with dependent-care responsibilities may be disproportionately affected (61, 62).

## 6 Bridging mechanisms from exposure to multisystem morbidity

### 6.1 Sleep disruption and circadian misalignment

Sleep disruption is a central bridging mechanism in the framework. It is not merely an outcome of shift work; it is also a pathway through which occupational exposures may influence psychological distress, physiological dysregulation, safety performance, and cardiometabolic risk. Studies report poor sleep quality and shortened sleep duration among COs, and shift work directly contributes to circadian misalignment (30, 31). Shift-worker cohort studies also highlight sleep-related factors relevant to insomnia-oriented occupational interventions (63). In correctional settings, sleep disruption may arise from night and rotating shifts, mandatory overtime, stress-related arousal, trauma exposure, and persistent hypervigilance.

The importance of sleep lies in its cross-domain effects. Insufficient sleep can impair cognitive function, emotion regulation, and vigilance, while circadian disruption is linked in the broader literature to obesity and cardiometabolic dysfunction (40, 64–67). CO-specific evidence increasingly connects sleep problems with mental disorder symptoms (40). Thus, sleep should be measured and managed as a safety-critical and public health outcome, not only as a personal wellness issue.

### 6.2 Chronic stress, allostatic load, and physiological dysregulation

Chronic stress provides a second bridge between correctional work and multisystem morbidity. Repeated stress-system activation may contribute to hypothalamic-pituitary-adrenal axis dysregulation, autonomic imbalance, inflammation, blood pressure elevation, and metabolic disruption (42, 43, 68). Allostatic load theory is useful because it explains why psychological distress and physical health problems may co-occur in the same workforce. Correctional work repeatedly activates vigilance, threat appraisal, emotional suppression, and institutional control demands, while long shifts and inadequate recovery may limit physiological restoration.

Direct biomarker evidence in CO populations remains limited, but the mechanistic logic is strong and testable. Future studies should include objective indicators such as heart rate variability, salivary cortisol, blood pressure, waist circumference, glycated hemoglobin, lipids, C-reactive protein, actigraphy-based sleep, and wearable sensor-derived fatigue metrics. Such measures would allow researchers to examine whether organizational exposures predict physiological dysregulation and whether integrated interventions reduce both psychological and biological burden.

### 6.3 Hypervigilance, coping, and lifestyle deterioration

Persistent hypervigilance is both an occupational adaptation and a health risk. Within correctional institutions, constant alertness helps officers detect threat. Outside work, however, sustained arousal may impair sleep, increase irritability, contribute to family conflict, and reinforce trauma-related symptoms (22, 69, 70). Stress-related coping behaviors may further connect occupational strain to physical health outcomes. Low physical activity, smoking, alcohol use, emotional eating, and disrupted meal timing can provide short-term relief but increase long-term cardiometabolic risk (19, 39, 71).

## 7 Downstream mental and physical health outcomes

### 7.1 Mental health burden

Mental health evidence among COs is comparatively mature. Chronic occupational stress is widely reported, and burnout is especially salient, with emotional exhaustion closely linked to work overload, inadequate recovery, low support, and organizational unfairness (13, 14, 18–24). Depression, anxiety, and broader psychological distress are also elevated across diverse correctional systems, with work-family conflict, violence exposure, low control, and institutional culture repeatedly implicated (15, 16, 59, 69, 72–74).

Trauma exposure and PTSD represent another established domain. COs may witness violence, self-harm, suicide attempts, medical emergencies, and direct assault (1, 15, 16). Emerging research also highlights moral injury, reflecting distress associated with institutional practices, role conflict, perceived betrayal, or inability to act in accordance with personal values (55, 56). Comparative evidence further suggests that lifetime versus work-related trauma, organizational commitment, and cynicism may differ between uniformed and nonuniformed correctional staff (75). Suicide risk and help-seeking barriers require particular attention because stigma, confidentiality concerns, masculine occupational norms, and distrust of services can delay care (26–29, 70, 76–78).

### 7.2 Emerging physical and behavioral health burden

Physical health evidence is less developed than mental health evidence but is increasingly important. Studies report elevated hypertension, obesity, central adiposity, and other cardiovascular risk factors in some correctional samples (32–34). These risks likely reflect interactions among chronic stress, sleep disruption, poor dietary patterns during shifts, low physical activity, and cumulative schedule burden. Causal inference remains limited, but available evidence supports treating cardiometabolic health as part of correctional occupational health surveillance rather than as a separate lifestyle issue.

Musculoskeletal disorders are also common. Officers may experience prolonged standing, walking patrols, static surveillance, sudden forceful movements during emergencies or restraints, and load carriage from duty equipment (35–37). Low back, neck, shoulder, and knee pain can contribute to disability, absenteeism, presenteeism, and early retirement (38). These outcomes demonstrate why integrated prevention must include ergonomic risk reduction and modified-duty planning in addition to mental health programming.

## 8 Intervention and policy levers

### 8.1 Individual-level strategies: useful but insufficient

Individual-level interventions include stress management, mindfulness, resilience training, physical activity promotion, sleep hygiene, and digital mental health tools (4, 45); evidence from adjacent public safety populations, including mindfulness-based programs among police officers, also suggests potential applicability to correctional settings (79). These approaches may improve coping, mental health literacy, and symptom management. However, their effects may be heterogeneous and difficult to sustain when the upstream work environment remains unchanged. The key limitation is not that individual interventions are irrelevant; it is that they are often asked to compensate for organizational hazards that exceed individual control. A recent systematic review and meta-analysis of wellbeing interventions among COs reported small-to-moderate effects on psychological outcomes, with effect sizes varying substantially by intervention type and outcome domain (45). These findings support the utility of individual-level approaches while highlighting the importance of tailoring and sustained implementation conditions.

### 8.2 Peer/team supports and culturally acceptable access to care

Peer support and team-based approaches may be more acceptable in correctional settings because they leverage shared occupational identity and reduce stigma (46, 80). However, one-off critical incident debriefing should not be conflated with comprehensive peer support or psychologically informed crisis support. Peer programs require training, supervision, confidentiality protections, referral pathways, and organizational legitimacy. Without these conditions, peer support risks becoming informal emotional labor rather than a sustainable health system component.

### 8.3 Organizational redesign and public health governance

Organizational strategies address upstream determinants. These include reducing mandatory overtime, increasing schedule predictability, applying circadian principles to roster design, strengthening staffing adequacy, improving supervisory support, institutionalizing procedural justice, and building confidential correctional-specialized mental health services (3, 25, 49, 52). Such measures are not simply administrative improvements; they are public health interventions because they change exposure intensity, recovery opportunities, and access to care. The relative advantage of organizational strategies over stand-alone individual programs is a theoretically grounded expectation consistent with the exposure structure of correctional work; head-to-head comparative trials remain scarce and represent a priority for the field.

### 8.4 Total worker health for correctional settings

The TWH approach provides a practical organizing framework because it integrates occupational safety and health protection with health promotion (12, 81, 82). In correctional settings, TWH should not be reduced to wellness education. A correctional TWH model would combine injury prevention, violence prevention, staffing and schedule reform, sleep and fatigue management, cardiometabolic screening, ergonomic risk reduction, confidential mental health care, peer support, and participatory implementation. Correctional employees also report organizational and environmental obstacles to healthy living that participatory programs must address (83). The Health Improvement through Employee Control program and related participatory approaches show how correctional workforces can be engaged in designing feasible interventions (82, 84). Some components of the proposed correctional TWH model are supported by correctional workforce studies, whereas others are extrapolated from broader occupational health and public safety literatures and require direct evaluation in correctional settings.

## 9 Evidence maturity and future research agenda

The evidence base is strongest for organizational stressors, burnout, psychological distress, trauma-related symptoms, and sleep problems. Evidence is moderate for violence-related trauma pathways, shift work-related sleep disruption, and musculoskeletal burden, whereas cardiometabolic pathways, individual wellness interventions, and organizational/TWH-informed interventions remain emerging. Table 3 summarizes the evidence maturity and research priorities across major occupational risk pathways.

**Table 3:** Evidence maturity and research priorities across occupational risk pathways.

Pathway	Evidence maturity	Current limitation	Research priority
Organizational stressors → burnout and psychological distress	Established	Most studies are cross–sectional; mechanisms linking staffing, schedules, organizational justice, and leadership to mental health trajectories remain under–tested.	Conduct longitudinal mediation studies linking organizational exposures to burnout, depression, anxiety, and psychological distress.
Violence and threat exposure → PTSD, hypervigilance, and moral injury	Moderate	PTSD evidence is comparatively stronger, whereas moral injury and hypervigilance are less consistently measured across correctional officer studies.	Use standardized instruments for trauma exposure, PTSD, moral injury, and hypervigilance in correctional officer cohorts.
Shift work and mandatory overtime → sleep disruption and fatigue	Moderate	Evidence supports sleep disruption and fatigue, but objective sleep measurement, roster–linked exposure data, and safety–performance outcomes remain scarce.	Combine actigraphy, roster data, wearable sensors, fatigue metrics, and safety–performance indicators.
Sleep disruption and chronic stress → cardiometabolic risk	Emerging	Correctional officer–specific longitudinal biomarker evidence is limited, and causal pathways remain insufficiently tested.	Establish cohorts measuring sleep, blood pressure, waist circumference, lipids, HbA1c, cortisol, HRV, and inflammatory markers.
Physical work demands → musculoskeletal pain and disability	Moderate	Musculoskeletal symptoms are documented, but ergonomic exposure assessment and intervention evidence in correctional settings remain limited.	Conduct ergonomic assessments, modified–duty evaluations, and prevention trials targeting low back, neck, shoulder, and knee pain.
Individual wellness interventions → mental health outcomes	Emerging	Existing intervention studies are heterogeneous, and evidence on durability, implementation quality, and correctional–specific adaptation remains limited.	Test comparative interventions with follow–up, implementation outcomes, and correctional–context adaptation.
Organizational and TWH–informed interventions → multisystem outcomes	Emerging	Rigorous evaluations of multilevel correctional interventions remain scarce, especially for combined mental, sleep, cardiometabolic, and organizational outcomes.	Use cluster randomized trials, stepped–wedge designs, or natural experiments to evaluate staffing, scheduling, leadership, and TWH–informed reforms.

Evidence maturity was classified as established, moderate, emerging, or plausible according to the volume, consistency, and correctional-officer specificity of available evidence. Established domains were supported by multiple correctional officer studies or prior reviews; moderate domains by several correctional officer studies with methodological or contextual heterogeneity; emerging domains by limited but growing correctional officer-specific evidence. TWH, Total Worker Health; PTSD, post-traumatic stress disorder; HRV, heart rate variability; HbA1c, glycated hemoglobin.

## 10 Discussion

### 10.1 Main contribution

This review reframes CO health as a multisystem occupational morbidity pattern rather than a collection of separate mental and physical disorders. The integrated risk pathways framework makes three contributions. First, it links upstream occupational exposures to downstream outcomes through explicit bridging mechanisms. Second, it shows why sleep disruption and chronic stress/allostatic load should be central to future correctional occupational health research. Third, it aligns intervention design with the structure of risk by emphasizing organizational and TWH-informed strategies rather than stand-alone resilience programs. Unlike prior scoping reviews that primarily map psychological health strategies (1) or co-locate safety and health promotion activities (2), the present framework specifies the bridging mechanisms that connect upstream exposures to multisystem outcomes and stratifies evidence maturity across pathways, converting a fragmented mental-versus-physical literature into a single, testable, and intervention-aligned model.

### 10.2 Why physical health evidence lags behind mental health evidence

The evidence imbalance between mental and physical health likely reflects measurement feasibility and research priorities. Psychological outcomes can be assessed with self-report scales at relatively low cost, while cardiometabolic, biomarker, sleep, and ergonomic assessments require equipment, clinical measures, repeated follow-up, and institutional cooperation. Physical conditions also have longer latency periods, making longitudinal designs essential. Nevertheless, the physical health evidence should not be considered secondary. Cardiometabolic risk, musculoskeletal pain, sleep disruption, and fatigue affect workforce sustainability, safety performance, absenteeism, and early retirement.

### 10.3 Implications for public health and correctional administration

The framework suggests several practical implications. Correctional systems should expand occupational health surveillance beyond injury reporting to include mental health, sleep quality, fatigue, cardiometabolic risk, and musculoskeletal complaints. Surveillance should be stratified by gender, rank, institutional security level, shift schedule, and exposure profile. Administrators should treat excessive overtime, unpredictable schedules, poor supervisory support, and inadequate recovery as modifiable exposure conditions rather than unavoidable features of correctional work.

Implementation should be participatory. Officers are more likely to engage with interventions that reflect correctional realities, protect confidentiality, and address operational constraints. Standing wellness committees involving administration, frontline officers, labor representatives, occupational health professionals, and mental health specialists may support feasible implementation. Public health agencies and correctional administrations should also consider cross-jurisdictional indicators for correctional staff wellbeing, enabling benchmarking and policy accountability. Surveillance and intervention design should also attend to sex and gender. Female correctional officers may face distinct exposures, including sexual harassment, heightened work-family conflict, and underrecognized cardiovascular risk (32, 61), while occupational norms emphasizing toughness may differentially shape help-seeking across genders (26–28). Sex-stratified and gender-sensitive monitoring is therefore needed to avoid masking subgroup-specific risk.

### 10.4 Limitations

This review has limitations. It is a structured narrative review rather than a registered systematic review; therefore, it synthesizes themes and pathways rather than providing exhaustive evidence mapping, formal risk-of-bias assessment, meta-analysis, or GRADE certainty ratings. Although dual screening and transparent search logic reduce selection error, residual selection bias remains possible. The underlying evidence is heterogeneous in design, measurement, and occupational context, and is frequently cross-sectional, limiting causal inference and cross-jurisdiction generalization. Longitudinal cohorts with repeated objective assessment—including actigraphy-based sleep, wearable fatigue monitoring, and standardized biomarker panels—remain scarce in correctional officer populations, constraining mechanistic and intervention evaluation. Correctional systems differ substantially across countries and institutional types, so the framework should be interpreted as an adaptable public health model rather than a universal causal model; exposure intensity and intervention priorities should be calibrated to local staffing systems, legal mandates, institutional security levels, and occupational cultures. A substantial portion of higher-quality CO evidence originates from Anglo-Saxon jurisdictions (notably Canada and the United States) and from Chinese-language samples, while evidence from Africa, South Asia, and many European and Latin American systems remains limited; this geographic concentration may constrain generalizability and introduce citation clustering around a small number of research groups. Finally, several pathways in the framework—particularly biomarker-based allostatic load, wearable sensor surveillance, and organizational intervention trials—remain emerging and require stronger empirical testing; evidence from underrepresented correctional systems is needed.

## 11 Conclusion

Correctional officer health is a public health and occupational safety issue generated by interacting organizational, psychosocial, behavioral, and physiological pathways. The literature shows substantial burdens of burnout, depression, anxiety, PTSD, moral injury, suicide risk, sleep disruption, cardiometabolic risk, musculoskeletal pain, and lifestyle deterioration. These outcomes should not be treated as isolated problems or solely as individual coping failures. Sleep disruption, chronic stress, allostatic load, hypervigilance, and maladaptive coping provide plausible bridges between correctional work exposures and multisystem morbidity.

For research, the priority is to move from descriptive burden studies toward longitudinal, mechanistic, and intervention-oriented designs. For practice, correctional systems should integrate mental health support, sleep and fatigue management, cardiometabolic surveillance, ergonomic prevention, and confidential care. For policy, staffing adequacy, schedule predictability, procedural justice, and TWH-informed organizational reform should be considered core components of correctional occupational health. Translating this framework into measurable health improvements will require sustained investment, participatory implementation, and rigorous evaluation of multilevel interventions.
