# Supplementary material for: Beyond burnout: occupational risk pathways linking stress, sleep, and multisystem morbidity in correctional officers
Source: Front Public Health. 2026 Aug 4;14:1901314. doi: 10.3389/fpubh.2026.1901314 (PMC13481589; doi:10.3389/fpubh.2026.1901314)
Supplement: Supplementary file 1 [file Supplementary_file_1.docx]

Supplementary Material S1. Search strategy and study selection logic

| 1. Search date | Searches were updated through February 2026. |
| --- | --- |
| 2. Databases | PubMed, Web of Science, Scopus, PsycINFO, and CNKI. |
| 3. PubMed search string | ("correctional officer*" OR "correctional worker*" OR "prison officer*" OR "prison staff" OR "jail staff")  AND  ("occupational stress" OR burnout OR depression OR anxiety OR PTSD OR "post-traumatic stress" OR sleep OR fatigue OR cardiovascular OR metabolic OR musculoskeletal OR "health promotion" OR wellness OR "Total Worker Health") |
| 4. CNKI 中文关键词 | 监狱警察 OR 戒毒人民警察 OR 司法警察 OR 监狱民警  AND  职业压力 OR 职业倦怠 OR 心理健康 OR 抑郁 OR 焦虑 OR 睡眠 OR 心血管 OR 代谢 OR 肌肉骨骼 OR 健康促进 |
| 5. Selection summary | 405 records were screened on title and abstract (303 English-language; 102 Chinese-language). After title and abstract screening, 278 records were excluded (198 English-language; 80 Chinese-language), leaving 127 records for full-text assessment. At the full-text stage, 58 records were excluded for not meeting prespecified eligibility criteria (Section 2.2 of the main manuscript), and 69 sources were retained for narrative synthesis. |
