# Supplementary material for: Beyond burnout: occupational risk pathways linking stress, sleep, and multisystem morbidity in correctional officers
Source: Front Public Health. 2026 Aug 4;14:1901314. doi: 10.3389/fpubh.2026.1901314 (PMC13481589; doi:10.3389/fpubh.2026.1901314)
Supplement: Supplementary file 2 [file Supplementary_file_2.docx]

**Supplementary Material S2**

*Flow of records through the review (PRISMA-inspired adapted for narrative synthesis)*

*Mental and Emerging Physical Health Risks in Correctional Officers: An Integrated Occupational Risk Pathways Framework*

Shiqi Sun, Peng Xing, Yi Liu, and Dongming Jia

## **S2.1 Purpose of this flow diagram**

This diagram documents the flow of records through the review process described in Section 2 (Methods) of the main manuscript. Although the present article is a structured narrative review and not a systematic review, the diagram adopts the visual conventions of PRISMA 2020 to support transparency in identification, screening, eligibility assessment, and final inclusion. It is not intended to substitute for the procedural rigor expected of systematic-review reporting (e.g., PROSPERO registration, formal risk-of-bias assessment, certainty-of-evidence grading), as acknowledged in Section 5.1 of the main manuscript.

## **S2.2 Flow diagram**

## **S2.3 Notes on the diagram**

(a) Identification. Records were identified through searches of PubMed, Web of Science, Scopus, and PsycINFO for English-language literature, and CNKI for Chinese-language literature, updated through February 2026. The combined yield was 405 records (303 English, 102 Chinese). Reference lists of retained articles and pertinent prior reviews were also hand-searched backward to identify additional contextually relevant sources. These sources were considered during full-text reading and synthesis where appropriate.

*(b) Screening. All 405 records were screened on title and abstract by two independent reviewers (DJ and SX) against pre-specified inclusion criteria. A total of 278 records were excluded (English n = 198; Chinese n = 80) for the reasons listed: outside population scope (e.g., incarcerated persons rather than staff), irrelevant outcomes, non-empirical work (e.g., editorials, opinion pieces), or other public-safety samples (e.g., police, firefighters) without separable correctional officer data.*

*(c) Eligibility. All 127 records proceeded to full-text review by both reviewers. Of these, 58 records were excluded because they did not meet one or more prespecified eligibility criteria (Section 2.2 of the main manuscript): the most common reasons were absence of correctional-officer-specific data, irrelevant outcomes, and non-separable findings from other public-safety populations. The remaining 69 sources met eligibility criteria and were retained to inform the narrative synthesis.*

(d) Inclusion. All 69 sources retained from the systematic search informed the narrative synthesis. Additional references cited in the main manuscript were identified through targeted backward citation searches of retained articles and pertinent prior reviews; these supplementary sources cover methodological guidance (e.g., SANRA, review typologies), theoretical frameworks (e.g., allostatic load), and adjacent occupational health literatures not captured by the primary search strategy.

*(e) Disagreements. Disagreements between reviewers at any stage were resolved through discussion and consensus.*

## **S2.4 Reporting framework**

Reporting follows the Scale for the Assessment of Narrative Review Articles (SANRA), per Baethge C, Goldbeck-Wood S, Mertens S. SANRA—a scale for the quality assessment of narrative review articles. Research Integrity and Peer Review 2019;4:5. https://doi.org/10.1186/s41073-019-0064-8
